# Cost Effectivities Analysis of Perovskite Solar Cells: Will it Outperform Crystalline Silicon Ones?

**DOI:** 10.1007/s40820-025-01744-x

**Published:** 2025-04-15

**Authors:** Yingming Liu, Ziyang Zhang, Tianhao Wu, Wenxiang Xiang, Zhenzhen Qin, Xiangqian Shen, Yong Peng, Wenzhong Shen, Yongfang Li, Liyuan Han

**Affiliations:** 1 State Key Laboratory of Metal Matrix Composites, Shanghai Jiao Tong University, Shanghai, 200240 People’s Republic of China; 2 State Key Laboratory of Advanced Technology for Materials Synthesis and Processing, Wuhan University of Technology, Wuhan, 430070 People’s Republic of China; 3 Beijing National Laboratory for Molecular Sciences, CAS Key Laboratory of Organic Solids, Institute of Chemistry, Chinese Academy of Sciences, Beijing, 100190 People’s Republic of China; 4 Materials Genome Institute, Shanghai University, Shanghai, 200444 People’s Republic of China; 5 Institute of Solar Energy, Key Laboratory of Artificial Structures and Quantum Control (Ministry of Education), School of Physics and Astronomy, Shanghai Jiao Tong University, Shanghai, 200240 People’s Republic of China; 6 Xinjiang Key Laboratory of Solid State Physics and Devices, School of Physical Science and Technology, Xinjiang University, Urumqi, 830046 People’s Republic of China

**Keywords:** Perovskite, Manufacturing cost, Levelized cost of electricity

## Abstract

The commercialization of perovskite solar cells (PSCs) has garnered worldwide attention and many efforts were devoted on the improvement of efficiency and stability. Here, we estimated the cost effectivities of PSCs based on the current industrial condition. Through the analysis of current process, the manufacturing cost and the levelized cost of electricity (LCOE) of PSCs is estimated as 0.57 $ W^−1^ and 18–22 US cents (kWh)^−1^, respectively, and we demonstrate the materials cost shares 70% of the total cost. Sensitivity analysis indicates that the improvement of efficiency, yield and decrease in materials cost significantly reduce the cost of the modules. Analysis of the module cost and LCOE indicates that the PSCs have the potential to outperform the silicon solar cells in the condition of over 25% efficiency and 25-year lifetime in future. To achieve this target, it is essential to further refine the fabrication processes of each layer in the module, develop stable inorganic transport materials, and precisely control material formation and processing at the microscale and nanoscale to enhance charge transport.

**Supplementary Information:**

The online version contains supplementary material available at 10.1007/s40820-025-01744-x.

## Highlights

- Current manufacturing cost of perovskite solar modules is calculated as 0.57 $ W^−1^ much higher than that of the silicon solar cells.
- Cost Effectivities analysis indicates that materials cost shares 70% of costs, and capital cost and other cost share nearly 15%, respectively.
- The cost of perovskite solar modules has the potential to outperform crystalline silicon under conditions of 25% efficiency, lifetime of 25 years, and cost reduction of materials and equipment, etc.

**Supplementary Information:**

The online version contains supplementary material available at 10.1007/s40820-025-01744-x.

## Graphical abstract

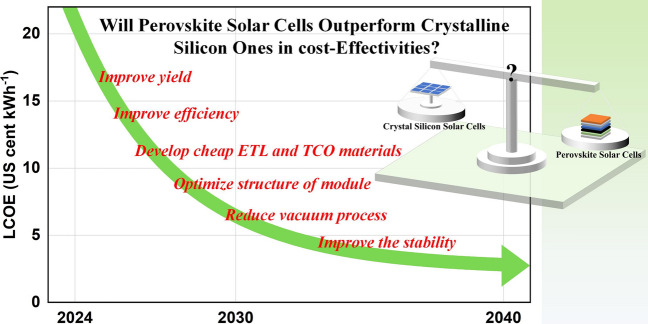

## Introduction

The growing demand for clean energy has driven a rapid expansion of the photovoltaic (PV) market, with the global solar PV capacity reaching over 400 GW in 2023, an increase in capacity over 80% compared to that in 2022 [1]. Crystalline silicon solar cells are currently the dominant player in the PV market, because of the excellent cost performance. With the growing demand for renewable energy by the development of human society, there is an expectation for the emergence of a new PV technology that is highly efficient and cost-effective. Perovskite solar cells (PSCs), as the next generation PV technology, have been receiving widespread attention since its appearance because of high efficiency and potentially low manufacturing cost [2].

In general, structures of the PSCs are classified based on whether the electron transport layer or the hole transport layer is located on the front electrode. Since the PSCs arisen from dye-sensitized solar cells [3], research of PSCs was initially focused on the regular structure [4, 5], with efficiency increased rapidly during the first decade [6, 7]. Inverted PSCs were first reported with a PCE of 3.8% in 2013 [8], and the first certified efficiency of 15% for the inverted PSCs with an aperture area > 1 cm^2^ was achieved in 2015 [9], through the development of heavy-doped NiO_x_-based hole transport layer, which greatly increased the influence of inverted PSCs. With the development of novel hole transport materials, interfacial regulation and surface modification [10–12], the efficiency gap between inverted and regular PSCs was considerably reduced, which further attracted greater attention in the solar cell community [13]. Furthermore, due to the wide application of self-assembled monolayers, the efficiency of inverted PSCs has significantly improved [14–18]. The champion efficiency of 27.0% by inverted PSCs was reported in 2025 [19]. Hence, the inverted PSCs have become leading research focus in both academy and industry. The progress was also made in the enlarging perovskite solar modules (PSMs). The first certified efficiency of PSM was achieved in 2016 with the efficiency of 12.1% [20], and the champion efficiency of mini-module is currently reported to 22.6% [21]. On the other hand, perovskite solar companies have been focusing their efforts to improve the efficiency and lifetime of large-area PSMs, and the PSMs with inverted structure also became the major type in the industry, especially in China. Currently, the module with a size of 1200 cm^2^ have achieved a steady-state efficiency of 19.2% efficiency [22], and the module with the commercial size (2 m^2^) also achieved an efficiency of 19.04% [23].

Long-term stability is another challenge issue for the commercialization of PSCs, which can be attributed to the ion migration, phase separation, poor interface stability and the stresses from the ambient environment, especially from the oxygen and moisture. So far, many studies have focused on addressing the stability issues above. For example, creating a depletion region with the perovskite layer to confine the mobile ions was reported as a promising strategy to inhibit the migration of iodide ions [24]. Also, the ion migration and phase separation can be suppressed by the homogenization of the perovskite component distribution [25]. Replacing the organic components in the devices with inorganic ones, such as using NiO_x_ as a hole transport layer and passivating the surface defects by aluminum oxide prepared by atomic layer deposition (ALD) [26, 27], can significantly improve the stability and enhance the interface strength. Improvement of encapsulation technology is an effective method to protect devices from oxygen and moisture [28]. At present, it has been reported that the PSCs passed the IEC 61215:2016 standards under over 9,000-h operational tracking [29]. Moreover, the advancements in stability achieved in the laboratory have attracted the attention from the industry toward PSCs. The PSMs manufactured by the perovskite companies have also made progress in stability, for example, several Chinese companies announced their modules have also passed the test by the IEC 61215 standards [30].

Besides the efficiency and stability, the cost of PSCs is also a critical issue for their commercialization. Unfortunately, there were a few studies on the cost analysis of PSCs. In 2017, Cai et al. firstly reported the module cost and LCOE, indicating that the module cost and LCOE are 0.21–0.28 $ W^−1^ and 3.5–4.9 US cents (kWh)^−1^, respectively, which is much lower in comparison with the crystalline silicon modules at that time [31]. In addition, Li et al. reported that the manufacturing cost of PSMs was 0.17 $ W^−1^ [32]. The conclusions of other previous works are similar, suggesting that the PSCs have potentially lower costs than that of the crystalline silicon cells [33–38]. However, these estimated results were considered to lack accuracy as the manufacturing process of PSMs was uncertain at that time.

During the past 7 years, there has been significant progress in both silicon and perovskite PV technologies. In 2023, the cost of the crystalline silicon modules has decreased largely with the latest price falling to 0.1 $ W^−1^, corresponding to a decrease of over 75% compared to that in 2017 [39–41]. On the other hand, many startup companies have emerged to manufacture PSMs around the world. Especially, there currently are over 100 perovskite companies in China, and several Chinese companies announced the installation of 100 MW manufacturing lines, and started to supply modules for demonstration, which indicates that the manufacturing process is established. Therefore, it is time to re-evaluate the module cost because the more accurate data of the materials and equipment prices are available.

Here, we estimated the cost of PSMs as well as LCOE based on 100 MW year^−1^ manufacturing capacity. Sensitivity analysis is also carried out, which reveals the impact of various factors on the module cost. Based on the sensitivity analysis, we proposed the cost target of the perovskite PV in the short-term and long-term future, and provided some research issues for these targets, which indicates that the PSCs have the potential to outperform the silicon solar cells in the condition of over 25% efficiency and 25-year lifetime by promoting the innovative technology and basic research.

## Assumptions

**Fig. 1:**
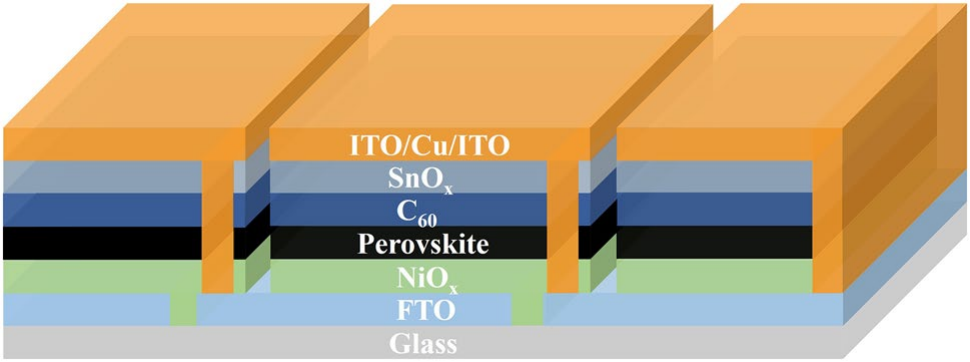
Schematic diagram of the perovskite solar module. For the cost analysis, the thickness of the film including NiO_x_, perovskite, C_60_, SnO_x_, ITO and Cu are 15, 600, 30, 10, 300 and 150 nm, respectively

To ensure the accuracy and relevance of the cost analysis, our assumptions were based on current industry trends and market research. There are over 100 perovskite solar companies in China, with ca. 20 of them having established 100 MW manufacturing lines. Reliable data including material and equipment costs are available for these manufacturing lines, making the 100 MW manufacturing line in China a suitable basis for estimating manufacturing costs. For the architecture, we selected the inverted PSM structure, as shown in Fig. 1, which is widely implemented in 100 MW year^−1^ manufacturing lines of Chinese perovskite solar companies. A similar structural design has also been adopted by 90% of perovskite companies in China, further demonstrating its feasibility and strong industry recognition. Regarding efficiency, while some companies reported module efficiencies of 19%-20%, these modules generally face stability challenges. Alternatively, modules with a size of 0.6 × 1.2 m^2^ and an output of 110 W (corresponding to an efficiency of approximately 15%), have been showcased at trade exhibitions and are commercially available in the Chinese market. Therefore, making the analysis on a 15% efficiency more accurately reflects the current situation [42]. With respect to yield, although companies did not share detailed yield data, private communications indicated that yields generally range from 30% to 60%. Hence, we set the yield at 50% in the following estimation. As for stability, given that the stability of perovskite solar cells has not yet been fully resolved, we estimated a lifetime of 5 years for the analysis.

**Fig. 2:**
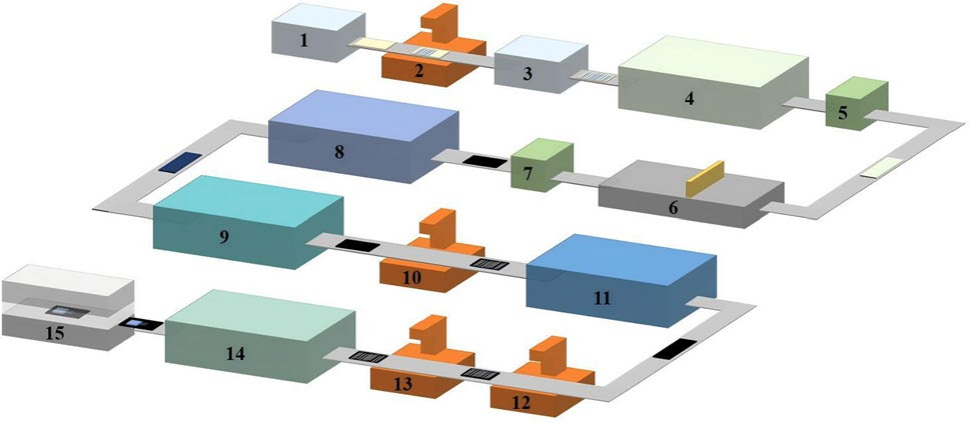
Process flow diagram of manufacturing for perovskite solar modules. (1. FTO glass clean before P1 laser scribing; 2. P1 laser scribing; 3. FTO glass clean after P1 laser scribing; 4. Deposition of NiO_x_ by magnetron sputtering; 5. Activation of NiO_x_ by UV/ plasma; 6. Deposition of perovskite by slot-die coating; 7. Vacuum dry; 8. Deposition of C_60_ by thermal evaporating; 9. Deposition of SnO_x_ by reactive plasma deposition (RPD); 10. P2 laser scribing; 11. Deposition of Cu by thermal evaporating and ITO by magnetron sputtering; 12. P3 laser scribing; 13. P4 laser edge cleaning; 14. Encapsulation; 15. Test.)

Figure 2 shows the manufacturing process of the PSMs, which can be divided into 15 steps. The main process includes the cleaning of fluorine-doped tin oxide (FTO) glass, the deposition of film such as NiO_x_, perovskite, C_60_, SnO_x_, and the composite electrode composed of Cu and indium tin oxide (ITO), laser scribing, encapsulation, and performance testing. Among the films, except for the perovskite film that is deposited by slot-die coating, the other films are deposited through physical vapor deposition (PVD) using the vacuum equipment.

## Result and Discussion

### Manufacturing Cost of PSMs

In general, the manufacturing cost can be divided by the materials cost, capital cost and other related costs including electricity, labor, land rent and maintenance.

Table 1 shows the various factors used to estimate material costs during the PSM manufacturing process, including material consumption, unit price, and utilization rate. Based on these factors, we estimated the material cost for the module with a size of 1 m^2^ to be approximately 29.3 $ m^−2^. And the corresponding materials cost was calculated to be about 0.39 $ W^−1^ in the condition of the efficiency of 15% and the yield of 50%.

**Table 1:** Consumption and price of perovskite solar module materials (1 m^2^)

Materials	Consumptions	Price	Utilization rate	Cost ($ m^−2^)
FTO Glass^1^	1 m^2^	10.0 $ m^−2^	0.98	10.2
NiO_x_^2^	0.015 cm^3^	19.0 $ cm^−3^	0.80	0.356
Perovskite^2^	2.83 g	0.63 $ g^−1^	0.95	1.89
C_60_^2^	0.0504 g	21.4 $ g^−2^	0.50	2.16
SnO_x_^2^	0.01 cm^3^	24.3 $ cm^−3^	0.80	0.304
ITO^2^	0.3 cm^3^	9.00 $ cm^−3^	0.80	3.38
Cu^4^	8 g	0.01 $ g^−1^	0.25	0.320
polyolefin elastomer (POE)^3^	1 m^2^	1.93 $ m^−2^	0.98	1.97
Back Glass^1^	1 m^2^	1.43 $ m^−2^	0.98	1.46
Al Frame^4^	1555 g	2.71 $ kg^−1^	0.98	4.30
Butyl rubber^3^	10.5 g	2.86 $ kg^−1^	0.98	0.0306
Junction Box^3^	1	2.86 $	0.98	2.92
Total				29.3

^1^Prices are sourced from Shandong Jinjing Science & Technology Stock Co., Ltd. ^2^Prices are sourced from the materials companies including Xi’an Yuri Solar Co., Ltd., Sigma-Aldrich (Shanghai) Trading Co., Ltd, Beijing Gaoke New Materials Technology Co., Ltd. and Shanghai Titan Scientific Co., Ltd. ^3^Prices are sourced from current market conditions. ^4^Prices are sourced from the website of Shanghai Metals Market (smm.cn)

**Fig. 3:**
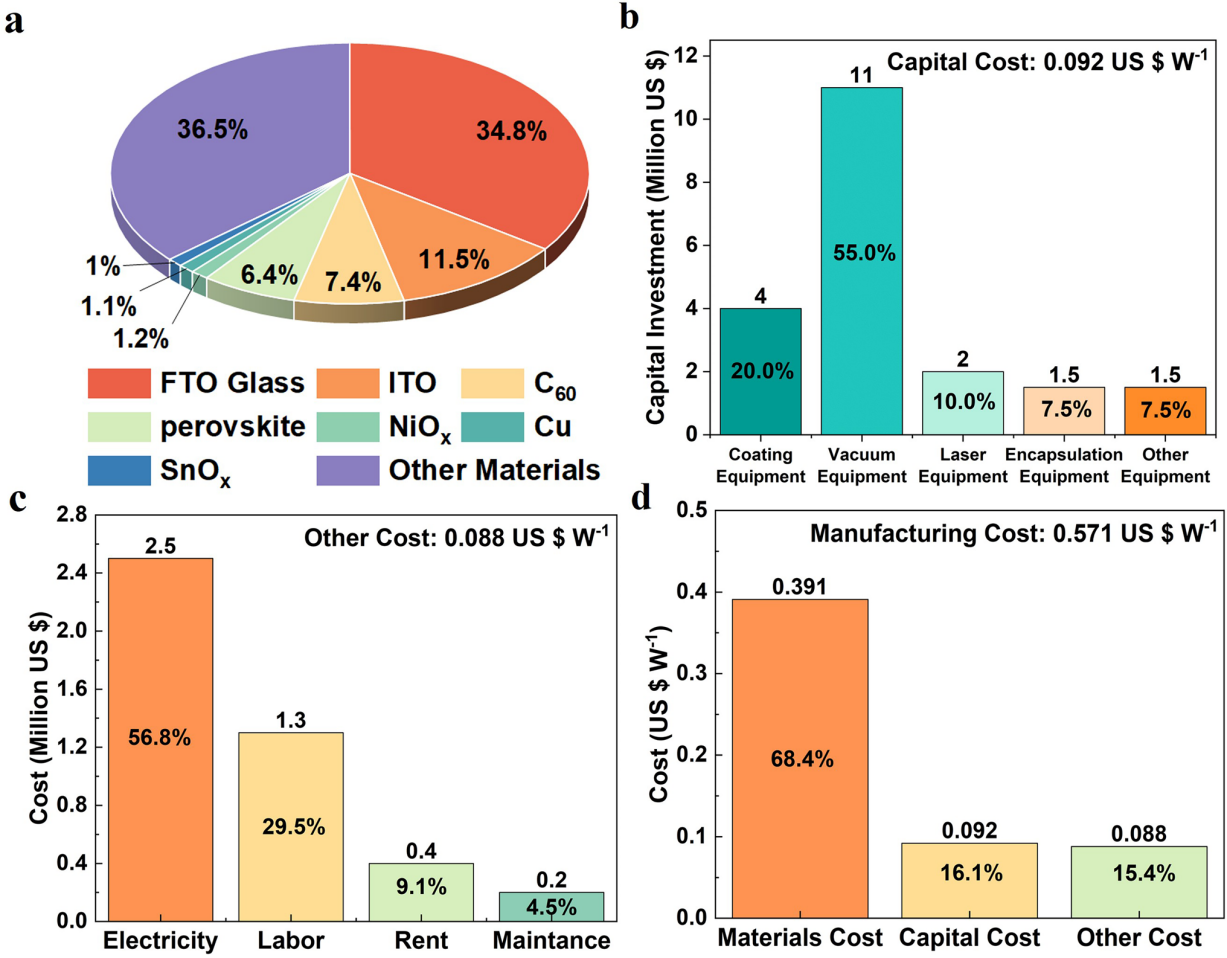
Breakdown of manufacturing cost for 100 MW PSM manufacturing line. **a** Material cost breakdown (“Other materials” include POE, back glass, aluminum frame, butyl rubber, and junction box). **b** Capital investment breakdown for manufacturing equipment (Other equipment includes conveyor belts, robotic arms, testing equipment, etc.). **c** Breakdown of other cost associated. **d** Overall manufacturing cost breakdown

Figure 3a shows the breakdown of materials cost. Although the “other materials” contribute a proportion of 36.5% totally, the details of them did not be discussed because the usages and prices of “other materials” are the same as traditional solar cells. Except for “other materials,” the FTO glass is the most expensive one, sharing the 30% of the total materials cost, followed by ITO and C_60_ occupying the share of 11.5% and 7.4%, respectively. In all, these three materials account for over half of the total materials cost. Notably, the cost of the perovskite layer itself contributes only about 6%, with less effect to the total materials cost.

Then, the breakdown of equipment investment is illustrated in Fig. 3b. The establishment of a manufacturing line with 100 MW capacity should be invested ca. 20 million USD by the information from equipment manufacturers and perovskite PV companies. Among the equipment, the investment of vacuum equipment occupies the largest share of over 50%, because of its high price and much more vacuum processes used in the production process. The costs of coating equipment and laser equipment occupy the share of 20% and 10%, respectively, with the second and the third highest cost share. In consideration of the 5-year depreciation period of equipment, the capital cost was calculated based on the amortization of the equipment investment [31]. As a result, the capital cost was estimated to be approximately 0.092 $ W^−1^.

Figure 3c shows the total expenses for electricity, labor, land rent and maintenance, which is calculated to be approximately 0.088 $ W^−1^. Among these, electricity costs account for nearly 60% of the total cost, being the largest expense. This is due to an excessive reliance of vacuum equipment in the manufacturing process, which consumes a large amount of electricity (450 KW for each vacuum machine) when the manufacturing line is in full operation.

The proportions of materials cost, capital cost and the other cost are shown in Fig. 3d. Notably, materials cost contributes nearly 70% of the manufacturing cost, while capital cost and the other cost are nearly equal, each around 15%. The total manufacturing cost of the module was estimated to be 0.57 $ W^−1^, much higher than that of the crystalline silicon module. The main reason for high cost calculated is the low efficiency and yield.

The module efficiency and yield will be improved in the future with the development of manufacture technology, and the prices of materials and investment will be expected to be reduced as the manufacturing capacity increase. Therefore, sensitivity analysis was performed in order to find out how to reduce cost in the future.

### Sensitivity Analysis

**Fig. 4:**
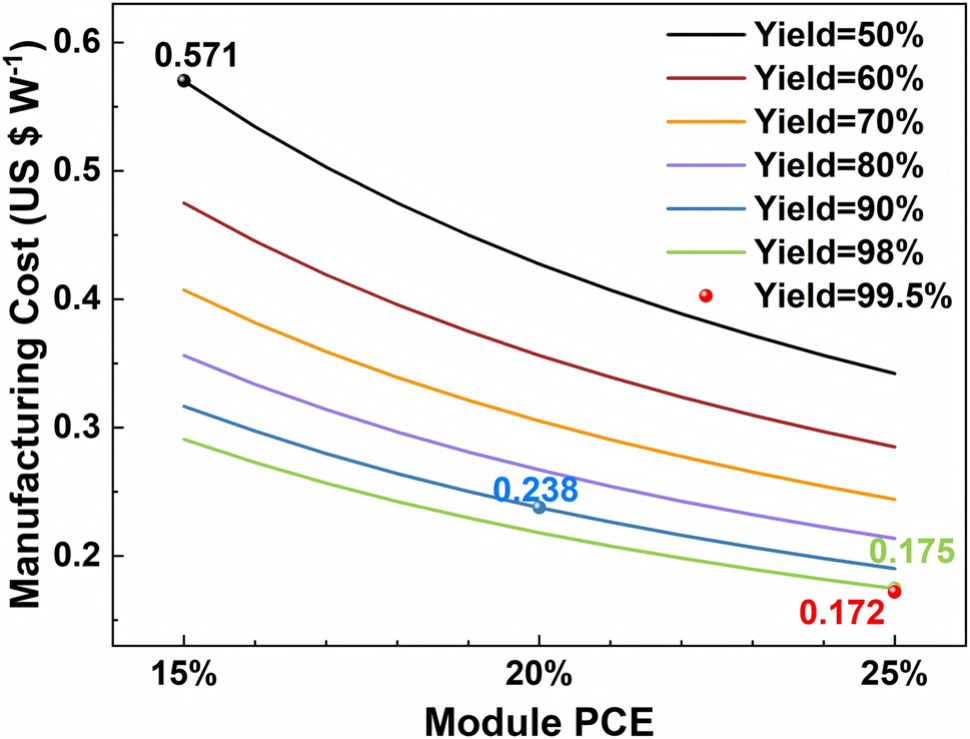
Sensitivity analysis of manufacturing cost with respect to PCE and yield

The effect of the efficiency and the yield on manufacturing cost is shown in Fig. 4. It is found that improving both the efficiency and the yield can reduce the cost of PSMs. Especially with the low module efficiency of 15%, improving the yield from 50% to 90% can significantly reduce the cost from 0.571 to 0.324 $ W^−1^, while only half effect of the cost reduction was obtained in the efficiency of 25%. Similarly, when the yield is low, improving the efficiency is more effective in reducing cost compared than the high yield condition. Even with the efficiency and the yield reaching 25% and 98%, respectively, the cost of PSMs (0.175 $ W^−1^) remains higher than that of the crystalline silicon modules (0.1 $ W^−1^). That means it is extremely difficult for PSMs to outperform the crystalline silicon modules in terms of the cost solely by improving the efficiency and the yield of PSMs at current technology level. Therefore, the impact of materials and equipment on the cost should also be taken into consideration.

For the convenience, we set three conditions for subsequently sensitive calculation. In relation with the current condition with an efficiency of 15% and a yield of 50%, the corresponding manufacturing cost is 0.571 $ W^−1^; the condition of scenario 1 is set as an efficiency of 20% and a yield of 90%, the corresponding manufacturing cost is 0.238 $ W^−1^; the condition of scenario 2 is set as an efficiency of 25% and a yield of 98%, the corresponding manufacturing cost is 0.175 $ W^−1^.

**Fig. 5:**
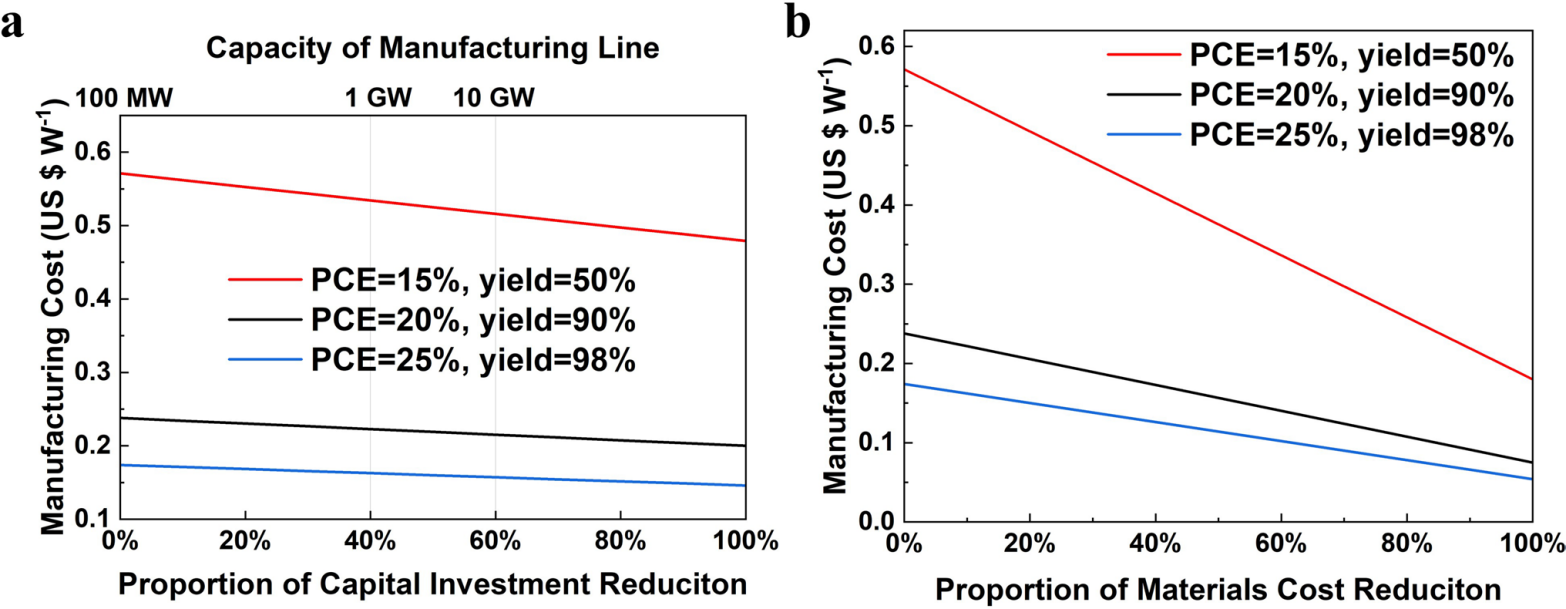
Sensitivity analysis of manufacturing cost. **a** The effect of capital investment on manufacturing cost. **b** The effect of materials cost on manufacturing cost

The relationship between the manufacturing cost and the equipment investment is shown in Fig. 5a. According to the estimates from the equipment manufacturers, as the manufacturing capacity expands, equipment investment per 100 MW can be reduced by about 40% when the capacity increases to 1 GW, and by 60%–70% when the capacity expands to 10 GW. Although reducing equipment investment has an effect on cost reduction, the effect is not significant because capital cost only shares a small proportion (ca. 15%) of the total cost. Even in the scenario 2 with 25% efficiency and 98% yield, the manufacturing cost is 0.14 $ W^−1^ still higher than that of the crystalline silicon modules. This means the PSMs cannot show advantage to the silicon solar modules after the depreciation of equipment finished (equipment investment is zero).

Figure 5b shows the sensitivity analysis of the materials cost. It is found that the decrease in the materials cost can effectively reduce the cost because it shares 70% of the total cost. However, for the current situation with 15% efficiency and 50% yield, even if the materials cost is reduced to zero, the remaining cost is 0.18 $ W^−1^, still higher than that of the crystalline silicon modules. For the purpose of reducing the cost of PSMs below that of the silicon solar modules, the materials cost should be reduced by 80% and 60% in the scenario 1 and scenario 2, respectively. Such a large reduction is obviously difficult to achieve. For example, the cost of the FTO glass shares the most proportion of 35% due to its high price of 10 $ m^−2^. According to the prediction by manufacturer, the price of the FTO glass is expected to decrease to 7 $ m^−2^ as the manufacturing capacity of PSMs scales up to 10 GW in the future. This price reduction of the FTO glass is not sufficient because which only contributes nearly 10% reduction in the materials cost. It is necessary to develop new transparent conductive substrates with lower costs. ITO used for rear electrode, sharing the second largest proportion, is commonly used in the manufacturing of various electronic devices such as liquid crystal display (LCD), organic light-emitting diode (OLED) and touch panel, so its price hardly decreases with the increase in the manufacturing capacity of PSMs. C_60_ is the third most expensive material, its price is difficult to be further reduced because of the limitation of its manufacturing process of discharging. One method to reduce the materials cost is to decrease the thickness of every layer, for example, the thickness of C_60_ can be decreased by accurately controlling the condition of thermal evaporation. Using cheap inorganic electron transport materials, such as SnO_2_ and TiO_2_, is also an effective way to reduce materials cost. Recently, SnO_2_ prepared by ALD was proposed to replace C_60_ [43], which indicates a possibility of reducing materials cost in future. Although there’re many research issues remained such as up-scaling, improvement of electron transport ability by doping method, developing new deposition process and optimizing the nano-layer structure of inorganic electron transport materials. Of course, it is necessary to develop more advanced inorganic electronic transport materials.

Based on this situation, the target cost of 0.1 $ W^−1^ is still difficult to achieve, even if in scenario 2. Therefore, we further adjusted and combined the factor of the cost elements, and found out in the condition of 25% efficiency, 99.5% yield as well as reductions of 40%, 50%, and 30% in materials, equipment, and electricity costs, respectively, the manufacturing cost will be reduced to about 0.1 $ W^−1^, almost equivalent to that of the crystalline silicon modules. We hence used this new condition as the scenario 2 for the following calculation of LCOE.

### Levelized Cost of Electricity

The LCOE is an average electricity generation cost, which reflects the cost performance of photovoltaic modules from another perspective, as the lifetime of modules is taken into consideration.

For the calculation of the LCOE for PSMs, we used the annuitization method [31, 44] as shown in Eq. (1):

1 $${\text{LCOE}} = \frac{{{\text{ICC}} \times 1000{\text{CRF}}}}{{{\text{CF}} \times 8760}} + {\text{O}}\& {\text{M}}$$

where ICC is the Installed Capacity Cost = MSP + BOS Cost;

*CRF* is Capital Recover Factor = (*i* × (1 + *i*)^*n*^)/((1 + *i*)^*n−1*^);

MSP is Minimum Sustainable Price [45];

BOS Cost is Balance of System cost;

*i* = discount rate;

*n* = lifetime of modules;

CF = Capacity Factor, CF is the ratio of actual energy generation to the maximum generation capacity;

O&M = Operation and Maintenance.

**Fig. 6:**
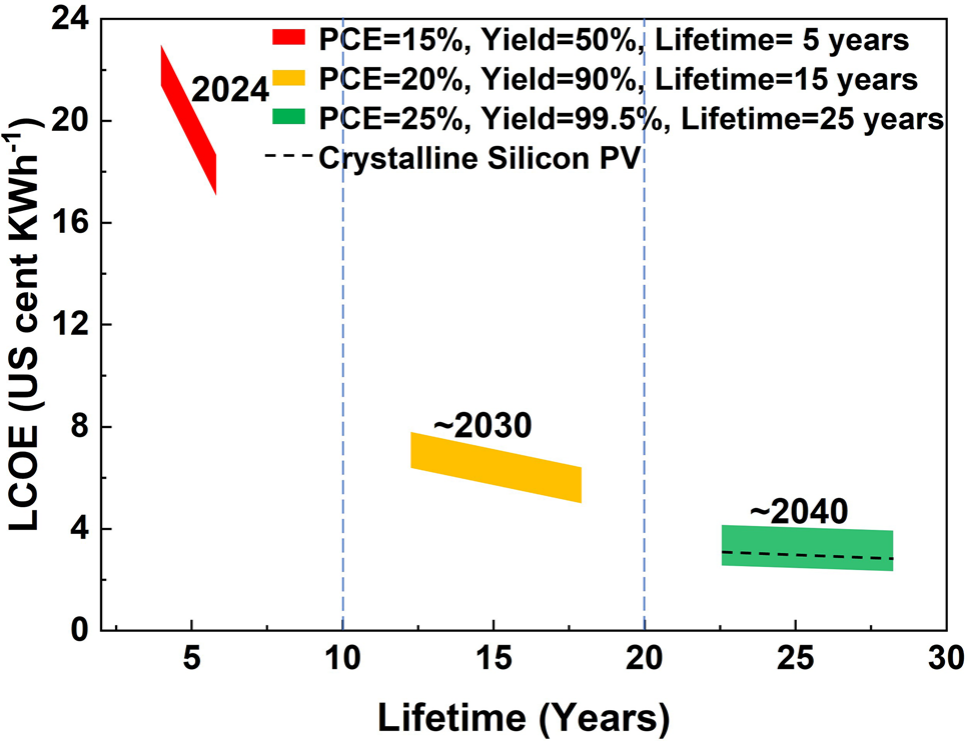
Estimated results of LCOE of PSMs in different stages

The LCOE of PSMs in different stages, which are divided by efficiency, yield and lifetime, is demonstrated in Fig. 6. The LCOE of current PSMs with the lifetime of 5 years is approximately 18-22 US cents (kWh)^−1^ (red area), which is 7 times higher than that of the crystalline silicon solar modules. As the power rate much lower than its LCOE, PSMs with this LCOE cannot be used for electricity generation because unprofitability, which may only be suitable for demonstration.

The LCOE of modules with 20% efficiency, 90% yield (scenario 1) and a lifetime of 15 years is 5–7 cents (kWh)^−1^ (yellow area), as the cost of the short-term target. From the progress of efficiency and stability, this target might potentially be achieved within a few years, because the module with a commercial size of 2 m^2^ was reported with a champion efficiency of 19.04% [23], although the reproducibility and stability of the module still need to be improved. Besides, many effective methods for improving stability have been proposed recently, such as passivating surface defects with ALD-deposited aluminum oxide [27], enhancing the stability of rear electrode with the composite electrode [46], and improving stability under reverse bias with a reinforced barrier of ITO/SnO_2_/C_60_/LiF [47]. Hence, this target of scenario 1 may be achieved in 4–5 years. Even if scenario 1 is achieved, it is still difficult to use PSMs for photovoltaic power station, because the LCOE of PSMs in scenario 1 is twice as high as that of crystalline silicon modules. However, the product in this stage can be considered to use in some specific market such as flexible and portable energy systems (wearable electronics and foldable solar chargers), power supply equipment for smart homes and PV system on the car roof in consideration of the advantages of PSCs such as lightweight, flexibility, high efficiency under low-light conditions, and customizable aesthetics.

For the scenario 2, it is necessary to obtain a yield of 99.5%, a module efficiency of 25% and a lifetime of 25 years in order to achieve a cost lower than that of crystalline silicon modules (green area, 3 US cents (kWh)^−1^). Although the current yield is approximately 50% or even lower due to limited experience in mass manufacturing, accompany with the manufacturing lines operating at full capacity, it is possible to improve the yield to over 99% by accumulating the manufacturing experience and optimizing the manufacturing process. The module efficiency of 25% requires that the PCE of small-area cells to exceed 28% due to the cell-to-module losses. To achieve the cell efficiency of 28%, an open-circuit voltage (*V*_OC_) of 1.23 V, a short-circuit current (*J*_SC_) of 27 mA cm^−2^ and a fill factor of 86% should be obtained. At present, these factors have been reported separately. The research issue is how to simultaneously achieve these parameters to realize the further improvement of PCE. One approach is to improve the capture rate of incident photon energy in the light absorbing material, that is, to increase the effective absorption of solar energy, to broaden the light absorption range. It requires the optimization of perovskite crystallization process to improve the quality of crystallization and purification of the components to adjust the perovskite bandgap so that it is close to the optimal photovoltaic bandgap [48]. The improvement of the light transmittance rate of the transparent conductive substrate is also required to alleviate the adverse effects brought about by the substrate. The other approach lies in attenuating the non-radiative recombination of photogenerated carriers, i.e., reducing the loss of the generated electrical energy inside the solar cell. For example, a) modulate the defect density of perovskite crystals, especially on the surface of crystals (~ 10^16^ cm^−3^), which is five orders of magnitude higher than that of perovskite single crystals (2 × 10^11^ cm^−3^), to inhibit defect-induced carrier recombination [10]; b) precisely control the thickness and deposition of the charge transport layer, optimize the structural design of the charge transport material, design and synthesis of functional charge transport materials at the nanoscale, thus to modulate the carrier mobility; c) develop nano-interface modified materials with multiple functions and optimize the interfacial contact between the charge transport material and the perovskite layer to optimize the carrier transport channel and inhibit carrier recombination [49]. Aforementioned methods need to be considered in a holistic manner, which may take a long time to solve the difficulties of simultaneous achieving these parameters. Recent efficiency progress indicates the efficiency of PSCs tends to be saturated, comparing to the 7.3 points improvement in efficiency (from 17.9% to 25.2%) between 2014 and 2019, while the efficiency only increased from 1.5 points (from 25.2% to 26.7%) between 2019 and 2024. Therefore, it is obvious that the further improvements in efficiency will become extremely difficult, which might take over a decade to achieve a cell efficiency of over 28%. Besides, although the highest cell efficiency is 27.0% now, the efficiency of the modules with ca. 1 m^2^ size is below 20%, the gap of the efficiencies is 7 points. Thus, how to reduce the cell-to-module losses is another research issue to obtain a 25% module efficiency. In addition, improving the lifetime from 10 to 25 years is a highly challenging task. It is difficult to achieve through improvements in current technologies and requires technological innovation. For example, the ion migration to outside can be blocked by passivation and the use of block layer [50, 51], while the phase separation caused by ion migration in perovskite layer will be more complex and requires new methods to address. When considering a degradation rate of 0.6% per year, the LCOE under scenario 2 will increase by 0.2 cents (kWh)^−1^. Therefore, it may take more than 15 years to achieve the target, which is likely to meet the time for mass production of PSCs proposed by some countries [52].

## Conclusion

**Fig. 7:**
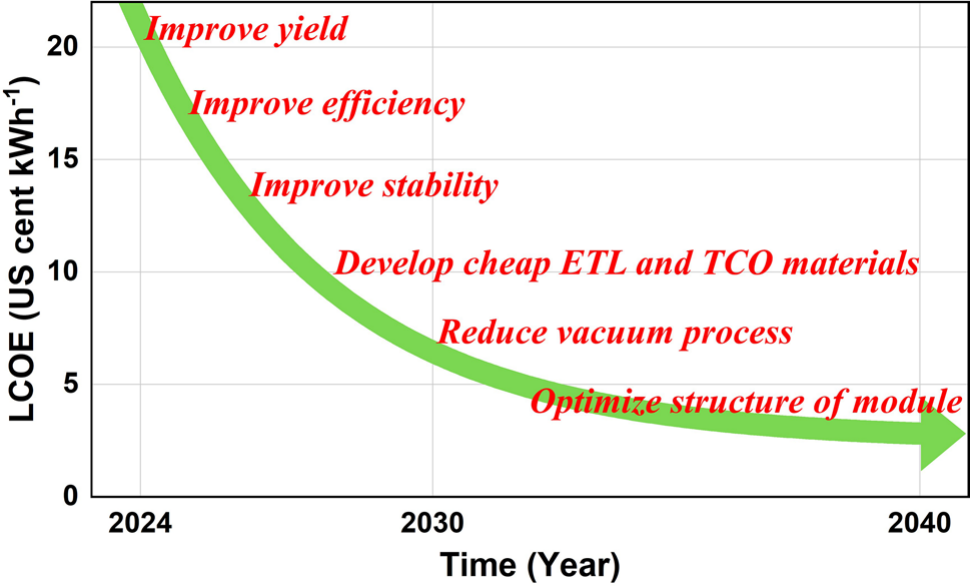
Roadmap of reducing the LCOE of PSMs to below that of silicon modules

The manufacturing cost of PSMs is calculated, based on the current manufacturing method. It is found that the materials cost contributes nearly 70% of the manufacturing cost, while capital cost and other cost are nearly equal, each around 15%. The manufacturing cost and LCOE of PSMs in 2024 were estimated as 0.57 $ W^−1^ and 18–22 US cents (kWh)^−1^, respectively. The sensitivity analysis indicates: (1) Improving the efficiency and yield is an urgent issue for the reduction of manufacturing cost; (2) The materials have a significant effect on the manufacturing cost, due to their large share of the total cost; (3) Reducing equipment investment is also required, which not only reduces the capital cost but also decreases the risk of the business. Hence, two scenarios are proposed for different development stages in future. Scenario 1 with 20% efficiency and 90% yield may be achieved in 4–5 years through the extension of current technologies. However, the modules at scenario 1 cannot be used for mass electricity generation market, because their module cost (0.24 $ W^−1^) is still higher than that of the silicon solar cells, so that it is necessary to seek new markets such as mobile electronic devices, toys, see-through devices, and indoor applications, etc., for the profitability. Scenario 2 shows the possibility that PSMs can obtain a cost similar to that of the crystalline silicon modules, under the coordination of over 25% efficiency, 99.5% yield, 40% materials cost reduction, 50% equipment investment reduction and 30% electricity cost reduction. Besides, the analysis indicates if PSMs could achieve a lifetime of 25 years, their LCOE could be equal to that of crystalline silicon modules (3 US cents (kWh)^−1^). Therefore, the conclusion can be drawn that PSCs can surpass the silicon solar cells when the module efficiency and the lifetime exceed 25% and 25 years, respectively. In conclusion, based on the above cost effectivities analysis, we proposed the roadmap for future direction with several examples of challenges in reducing the LCOE of perovskite modules (Fig. 7), such as to improve production yield, to improve module efficiency and stability, to develop cheap ETL and TCO materials, to reduce vacuum process, and to optimize structure of module. The achievements in such research topics will accelerate the progress in a more rapidly reducing cost to below that of silicon modules. As a micro–macro process, it is also important to develop high throughput processing technologies of PSMs if this PV technology is aiming for this purpose.

## Supplementary Information

Below is the link to the electronic supplementary material.

Supplementary file1 (DOCX 31 KB)
