# Supplementary material for: Cost Effectivities Analysis of Perovskite Solar Cells: Will it Outperform Crystalline Silicon Ones?
Source: Nanomicro Lett. 2025 Apr 15;17:219. doi: 10.1007/s40820-025-01744-x (PMC12000492; doi:10.1007/s40820-025-01744-x)
Supplement: Supplementary file 1 — Supplementary file1 (DOCX 31 KB) [file 40820_2025_1744_MOESM1_ESM.docx]

Supporting Information for

**Cost Effectivities Analysis of Perovskite Solar Cells: Will It Outperform Crystalline Silicon Ones?**

Yingming Liu^1^, Ziyang Zhang^1^, Tianhao Wu^4^, Wenxiang Xiang^1^, Zhenzhen Qin^1^, Xiangqian Shen^6^, Yong Peng^2^, Wenzhong Shen^5^, Yongfang Li^3^, Liyuan Han^1,^ *

^1^ State Key Laboratory of Metal Matrix Composites, Shanghai Jiao Tong University, Shanghai 200240, People’s Republic of China

^2^ State Key Laboratory of Advanced Technology for Materials Synthesis and Processing, Wuhan University of Technology, Wuhan 430070, People’s Republic of China

^3^ Beijing National Laboratory for Molecular Sciences, CAS Key Laboratory of Organic Solids, Institute of Chemistry, Chinese Academy of Sciences, Beijing 100190, People’s Republic of China

^4^ Materials Genome Institute, Shanghai University, Shanghai 200444, People’s Republic of China

^5^ Institute of Solar Energy, Key Laboratory of Artificial Structures and Quantum Control (Ministry of Education), School of Physics and Astronomy, Shanghai Jiao Tong University, Shanghai 200240, People’s Republic of China

^6^ Xinjiang Key Laboratory of Solid State Physics and Devices, School of Physical Science and Technology, Xinjiang University, Urumqi 830046, People’s Republic of China

* Corresponding author. E-mail: [han.liyuan@sjtu.edu.cn](mailto:han.liyuan@sjtu.edu.cn) (Liyuan Han)

**S1 Calculation of capital cost for 100 MW PSMs manufacturing line**

$\text{Capital Cost }\text{= }\text{Equipment Investment}\text{ ×}\text{CRF}$ (S1)

$\text{CRF }\text{= (}\text{i}\text{ × (1 + }\text{i}\text{)\textasciicircum n)/((1 + }\text{i}\text{)\textasciicircum n - 1)}$ (S2)

*CRF* is Capital Recover Factor,

*i* = discount rate,

*n* = equipment depreciation period.

Calculated at a discount rate of 5% and a depreciation period of 5 years;

Capital Cost = Equipment Investment × CRF = 20 million USD × 0.23 = 4.6 million USD. Therefore, the Capital Cost for 100 MW/year manufacturing line with the yield of 50% was 4.6 million USD.

**S2 Electricity cost of manufacturing line for PSMs**

We assumed that there are 10 days per year allocated for equipment maintenance, so the equipment operates for 355 days. Based on this operating time, we calculated the total electricity consumption of the equipment: 2700 KW×355×24 h ≈ 23 million kWh. In China, the power rate is about 0.11 $/KWh, leading to a total electricity cost of: 23 million KWh×0.11 $/kWh ≈ 2.5 million US $ per year

**S3 Rent cost of manufacturing line for PSMs**

Currently, there is the manufacturing lines for PSMs with the capacity of 100 MW/year occupying approximately 10,000 square meters [S1]. In China, the land rental cost is about 0.1 $/m^2^/day, which corresponds to an estimated annual rental cost of approximately 0.36 US million $ per year.

**S4 Labor cost of manufacturing line for PSMs**

In 2016, a 1 GW CIGS manufacturing line required 700 employees [S2], which corresponds to about 70 employees for a 100 MW manufacturing line. We assume that the number of employees on a manufacturing line for PSMs will be similar to that of the CIGS manufacturing line. Considering the increased level of automation over time, we have reduced the number of employees for a 100 MW manufacturing line for PSMs to 60. In consideration with the current average salaries of employees in the photovoltaic industry in China, we estimated that the labor cost would be approximately 1.3 million US $ per year.

**S5 Calculation of MSP (minimum sustainable price) for PSMs**

Manufacturing cost (MC) only represent the expenses incurred by manufacturers in producing photovoltaic modules and do not determine the price charged to customers or the market. The module price must include the MC, overhead costs (OH), and the manufacturer's profit margin (WACC, or weighted average cost of capital) [S3]. The calculation of the Minimum Sustainable Price (MSP) follows the formula:

$\text{MSP}\text{ = }\text{MC}\text{ ×}\text{ }(1+OH+WACC)$ (S3)

According to Bloomberg New Energy Finance [S4], for the Asia-Pacific region, the range of WACC is approximately 2% to 8% of the MC, hence we set the value at 6%. The average overhead cost (OH) typically accounts for about 10% of the MC.

**S6 Assumptions for LCOE Calculation**

$\text{LCOE}\text{ }\text{=}\text{ }\text{(}\text{ICC}\text{ }\text{×}\text{ }\text{1000}\text{ }\text{CRF}\text{ }\text{)/(CF}\text{ }\text{×}\text{ }\text{8760)}\text{ }\text{+}\text{ }\text{O\&M}$ (S4)

Where ICC is the Installed Capacity Cost = MSP + BOS Cost;

*CRF* is Capital Recover Factor = (*i* ×(1+ *i*)^*n* )/((1+ *i*)^*n* -1);

*i* = discount rate;

*n* = lifetime of the modules;

where *i* = 5%, and when lifetimes of the modules are set to 5, 15, and 25, the CRFs are 0.231, 0.096, and 0.071, respectively.

MSP is Minimum Sustainable Price, and calculation of MSP is shown in S5;

BOS Cost is Balance of System cost, the BOS cost of perovskite photovoltaics is shown in Fig.S1;

CF = Capacity Factor, CF is the ratio of actual energy generation to the maximum generation capacity, the CF of 13% is based on the equivalent sunshine duration of the location of Chinese perovskite solar cells company.

O&M = Operation and Maintenance, typically, O&M = 0.001 $/kWh.

**S7 Manufacturing process of perovskite solar modules**

According to the Fig. 2, in the manufacturing process of PSMs, the first step is to clean the FTO glass to remove the dust and oil. Then, FTO substrate is laser-scribed (P1) to separate subcells. After that, FTO glass needs to be cleaned again to remove the scrap and dust generated by P1. The hole transport layer (HTL) is deposited on precleaned FTO substrates by the magnetron sputtering of NiO_x_, and the NiO_x_ needs to be activated by UV/ plasma. The perovskite layer is deposited by slot-die coating the precursor solution of perovskite. After slot-die coating, vacuum drying is necessary to remove residual solvents and promote perovskite crystallization. The electron transport layer (ETL) is deposited by thermal evaporation of C_60_. The buffer layer is SnO_x_, which is deposited by reactive plasma deposition, a technique that enables high deposition rate, high uniformity, and little damage to the organic layer. Before electrode, the P2 lines were laser-scribed. The deposition of ITO/Cu/ITO composite electrode is achieved through thermal evaporation of Cu and magnetron sputtering of ITO. After the electrode deposition, P3 lines were laser-scribed and P4 edge were cleaned to complete the module fabrication.

**Table S1 Equipment Cost for perovskite Module Fabrication**

| Equipment | Number | Unit Price (Million $) | Power (KW) |
| --- | --- | --- | --- |
| Slot-die | 2 | 2 | 150 |
| PVD (evaporation) | 1 | 1.4 | 450 |
| PVD  (magnetron sputtering) | 2 | 2.8 | 450 |
| RPD | 1 | 4 | 450 |
| Laser | 4 | 0.5 | 20 |
| encapsulation | 1 | 1.5 | 250 |
| Other | 1 | 1.5 | 270 |
| Total |  | 20 | 2700 |

The price and power of the equipment are provided by the manufacturer.

**Supplementary References**

[S1] Mellow Energy Commences Construction Project for 100 MW Perovskite Solar Module Manufacturing Facility (2024). https://www.mellow-energy.cn/official/journalism/details/33/0/115.html

[S2] K. A. W. Horowitz, R. Fu, M. Woodhouse, An analysis of glass–glass CIGS manufacturing costs. Sol. Energy Mater Sol. Cells. **154**, 1-10 (2016). <https://doi.org/https://doi.org/10.1016/j.solmat.2016.04.029>

[S3] Solar Manufacturing Cost Analysis (2024). <https://www.nrel.gov/solar/market-research-analysis/solar-manufacturing-cost.html>

[S4] BloombergNEF Information (2022). <https://posts.careerengine.us/p/6385ee13542d7268c20dd245>
